# Reproductive Outcomes and Fertility Patterns in Women with Systemic Sclerosis: A Multicentre Observational Study

**DOI:** 10.31138/mjr.200825.ser

**Published:** 2025-12-31

**Authors:** S Chandrashekara, Padmanabha Shenoy, Alakendu Ghosh, Sapan Pandya, Uma Kumar, Apurva Khare, Rajkiran Dudam, Arghya Chattopadhyay, Rudra Prosad Goswami

**Affiliations:** 1 ChanRe Rheumatology and Immunology Centre and Research, Bengaluru, India;; 2 Centre for Arthritis & Rheumatism Excellence (CARE), Cochin, Kerala, India;; 3 Clinical Immunology & Rheumatology Institute of Post-Graduate Medical Education and Research, Kolkata, India;; 4 Rheumatic Disease Clinic, Vedanta Institute of Medical Science, Ahmedabad, Gujarat, India;; 5 Department of Rheumatology, All India Institute of Medical Science, New Delhi, India;; 6 Department of General Medicine, LN Medical College and Research Centre, Kolar Road, Bhopal, Madhya Pradesh, India;; 7 HRC Hospital, Prakash Nagar, Hyderabad, India;; 8 Clinical Immunology and Rheumatology Services, Department of Internal Medicine, Postgraduate Institute of Medical Education and Research, Chandigarh, India;; 9 Department of Rheumatology, AIIMS, New Delhi, India

**Keywords:** systemic sclerosis, female reproductive health, fertility, pregnancy complications, abortion, caesarean section

## Abstract

**Aim:**

Limited data are available from India to guide patient counselling and management in systemic sclerosis (SSc). This study evaluates reproductive patterns, fertility outcomes, and pregnancy complications before and after SSc onset, comparing married and unmarried women in India.

**Methods:**

Data were extracted from the Indian Rheumatology Association registry across seven centres. Female patients meeting ACR/EULAR 2013 criteria for SSc were included. Reproductive history, menstrual patterns, cumulative fertility rate (CFR), pregnancy wastage ratio, stillbirth, and abortion rates were analysed, with comparisons between married and unmarried women, and between pre- and post-disease pregnancies.

**Results:**

Of 183 patients, 131 women were analysed (117 married, 14 unmarried). Married women had later disease onset (37.44 ± 10.57 vs. 19.79 ± 3.69 years; P < 0.001) and more comorbidities (46.15% vs. 14.28%; P = 0.024), while menstrual irregularities were more frequent in unmarried women (28.57% vs. 11.11%). Post-disease pregnancies occurred at younger disease onset (25.44 ± 5.83 vs. 38.08 ± 10.42 years; P < 0.001). Parity, abortion, and live birth rates were similar between pre- and post-disease pregnancies, but vaginal deliveries predominated pre-disease (186 vs. 6), whereas caesarean sections were more frequent post-disease (4/10; P = 0.023). Overall CFR was 1.68, pregnancy wastage ratio 125/1,000 pregnancies, lifetime abortion rate 221.37/1,000 women, and stillbirth rate 30.53/1,000 women.

**Conclusion:**

Women with SSc exhibit reduced fertility and increased pregnancy loss. Early-onset disease affects marital prospects and reproductive potential. Multidisciplinary care with preconception counselling and vigilant pregnancy monitoring is essential to optimise maternal and foetal outcomes.

## INTRODUCTION

Systemic sclerosis (SSc) is a rare autoimmune rheumatic disease characterised by vascular dysfunction, immune activation, and progressive fibrosis of the skin and internal organs. A systematic review and meta-analysis published in 2021 analysed 61 studies estimated a pooled global SSc prevalence rate of 17.6 cases per 100,000 individuals.^1^ In India, the estimated prevalence is slightly lower, at approximately 12 cases per 100,000 population.^2^ The disease exhibits a marked female predominance, affecting women four to nine times more frequently than men, with disease onset occurring during the reproductive years in nearly 50% of cases.^3,4^ The interplay between SSc and reproductive health is multifactorial, as it can adversely impact fertility through disease activity, organ involvement, or immunosuppressive therapy.

The impact of SSc on fertility may be attributed to immune-mediated damage related to the disease itself or the gonadotoxic effects of immunosuppressive therapies. Autoimmune diseases like SSc can modulate reproductive physiology, leading to challenges in conception, pregnancy maintenance, and delivery outcomes. A significant proportion of women with SSc report menstrual irregularities, including oligomenorrhea, amenorrhea, and premature menopause. Furthermore, hormonal fluctuations during key reproductive milestones such as menarche, pregnancy, and menopause may influence autoimmune disease activity and clinical outcomes.^5,6^

Although pregnancy in women with SSc has become increasingly common, data on maternal and foetal outcomes in this population remain limited. Available evidence suggests a higher incidence of adverse outcomes, including preterm delivery and intrauterine growth restriction (IUGR), among women with SSc.^7–9^ A single-centre study by Barilaro et al. further highlighted elevated risks of miscarriage, preeclampsia, small for gestational age (SGA) newborns, and preterm birth in this patient group.^10^

Considering the limited data from the Indian population, this study aimed to address this gap by analysing reproductive health characteristics in women with SSc using multicentric data from the Indian Rheumatology Association (IRA) registry. It evaluated menstrual patterns, fertility rates, and adverse pregnancy outcomes in both married and unmarried women, and compared reproductive outcomes before and after the onset of the disease.

## METHODOLOGY

This independent, prospective, multicentre observational study enrolled female patients diagnosed with SSc according to the ACR/EULAR 2013 classification criteria across seven rheumatology centres in India.^11^

Both newly diagnosed and follow-up patients were included. Data were obtained from the IRA registry, a nationwide database initiated in April 2020 to capture demographic and clinical details of six autoimmune rheumatic diseases (AIRDs): rheumatoid arthritis, spondyloarthritis, psoriatic arthritis, systemic lupus erythematosus, Sjögren’s syndrome, and SSc. The participating centres were selected to ensure broad geographic representation and adequate patient volume. Structured proformas were used to collect clinical and reproductive health information, including demographic details, age at disease onset, socioeconomic background, obstetric, and gynaecological history, marital status, and disease-related characteristics. Clinical research associates (CRAs) completed these proformas during outpatient visits through direct patient interviews and review of clinical records. To ensure uniformity across centres, both CRAs and site investigators participated in centralised online training coordinated by the nodal centre. Any methodological queries during the study were addressed by the nodal principal investigator. Ethical approval was obtained from the Institutional Ethics Committees and Independent Ethics Committees of all participating centres, and written informed consent was secured from all patients. The study was approved under the following protocol numbers and dates: IEC-CRICR-132/101/2020 (05/10/2020), IEC-59/03.07.2020.RP-462020 (15/07/2020), IPGME&RC/IEC/2020/478 (16/06/2020), and LNMC&RC/Dean/2020/Ethics/146 (07/08/2020). As per the institutional guidelines, written informed consent was obtained from all enrolled subjects.

Reproductive health data included information on age at menarche, menstrual cycle characteristics, and menopausal status in older women. Pregnancy-related details such as gravida, parity, abortions, medical terminations of pregnancy (MTPs), and intrauterine deaths (IUDs) were documented separately for the periods before and after disease onset. Marital status, including any history of divorce, was also recorded. Following data extraction using the proformas, a data cleaning process was undertaken. Records with missing values in all fields, those lacking pregnancy outcome data or containing inconsistencies, and records without information on the duration of illness were excluded.

Reproductive health metrics were calculated using standard epidemiological formulas. These included the pregnancy wastage ratio, stillbirth rates, abortion rates, and cumulative fertility rates. The pregnancy wastage ratio was estimated as the number of stillbirths and abortions divided by the total number of pregnancies, multiplied by 1000.^12^ Stillbirth rates were computed both per 1000 live births and per 1000 pregnancies.^13,14^ Lifetime abortion rates per 1000 women were calculated separately for married women and the overall cohort. Abortion rates were expressed as the number of abortions per 1000 pregnancies, as well as lifetime abortion rates per 1000 women.^15^ The cumulative fertility rate (CFR) was calculated as the number of live births per woman.^16^

### Sample size calculation

For the comprehensive analysis of the six AIRDs, an estimated total sample size of 6,500 patients was determined, based on the reported prevalence of each condition.^17^ The sample size for each disease was allocated proportionally according to its individual prevalence. For SSc, with a reported prevalence of 12 cases per 100,000 individuals (0.00012), a 95% confidence level, and a precision (margin of error) of ±0.2% (d = 0.002) with an alpha value of 0.05, the required sample size was estimated using the formula n = (Z^2^ × p × (1−p)) / d^2^, where Z = 1.96 for 95% confidence, p = expected prevalence, and d = desired precision. Substituting the values, the numerator was calculated as 3.84 × 0.00012 × 0.999 = 0.00046, and dividing by d^2^ (0.002^2^ = 0.000004) yielded a sample size of 115.^2^

### Statistical analysis

Stratified analyses were conducted to compare reproductive outcomes before and after SSc onset, including pregnancy wastage ratio, stillbirth and abortion rates, case fatality rate (CFR), and pregnancy-related complications. The influence of marital status on reproductive health parameters was also evaluated. Data included variables such as age at recruitment, age at menarche, menstrual regularity, age at marriage, duration of marriage, probable illness duration, and age at disease onset.

Descriptive statistics summarised the data, with normally distributed continuous variables expressed as mean ± standard deviation (SD) and non-normally distributed variables as median with range. Categorical variables were presented as frequencies and percentages. The normality of continuous variables was assessed using the Shapiro–Wilk test (P > 0.05 indicating normal distribution; P ≤ 0.05 indicating non-normal distribution).

Comparisons between married and unmarried groups were performed using independent sample t-tests for normally distributed variables and the Mann–Whitney U test for non-normal variables. Categorical variables were analysed using Chi-square or Fisher’s exact test, as appropriate. For fertility and pregnancy outcomes among married women before and after disease onset, paired t-tests were applied for normally distributed variables and Fisher’s exact test for categorical variables. All analyses were performed using SPSS version 29.0.2.0 and Python version 3.8.3, and tables were generated using Microsoft Excel (version 2409, build 16.0.18025.20030).

## RESULTS

A total of 183 patients were recruited; 19 males were excluded, and of the 164 female patients, 33 were excluded due to unavailability of data. This resulted in a dataset of 131 female patients with SSc retrieved from seven centres using the database. Among these, 117 were married and 14 were unmarried.

The overall cohort of 131 female patients had a mean age at recruitment of 46.83 ± 12.14 years (range: 22–81 years; median: 47 years). The mean age at menarche was 13.72 ± 1.19 years (range: 11–17 years; median: 14 years). Regarding menstrual regularity, 105 participants reported regular cycles, 17 had irregular cycles, and 9 were not applicable.

The mean total probable duration of illness was 130.49 ± 83.69 months (range: 6–480 months; median: 117 months). The mean age at disease onset was 35.65 ± 11.41 years (range: 12–71 years; median: 34.5 years). The mean age at marriage among the cohort was 20.36 ± 3.18 years (range: 13–30 years; median: 20 years). In the overall cohort, the lifetime stillbirth rate was 30.53 per 1000 women, the lifetime abortion rate was 221.37 per 1000 women, and the CFR was 1.68.

### Fertility patterns and pregnancy outcomes among married and unmarried SSc patients

Married patients were significantly older at the time of recruitment than unmarried patients (48.74 ± 11.15 years vs. 30 [24–50] years; P < 0.001). The age at menarche was slightly earlier in unmarried women (13.14 ± 1.56 years) compared to married women (14 [11–17] years; P = 0.052). Menstrual irregularities were more common in unmarried women (28.57%) than in the married cohort (11.11%).

Married women had a longer probable duration of illness (111 [6–480] months vs. 117.5 ± 63.16 months; P = 0.573) and a significantly higher age at disease onset (37 [18–71] years vs. 19.79 ± 3.69 years; P < 0.001) than unmarried women. Comorbidities were also more frequent in married women (46.15% vs. 14.28%; P = 0.024) (**Table 1**).

**Table 1.** Comparison of various parameters between married and unmarried patients.

**Parameters**	**Married (n = 117)**	**Unmarried (n = 14)**	**P value**
**Age**	48.74 ± 11.15^b^	30 (24–50)^a^	<0.001^^^
**Age at menarche (n= 115)**			
	14 (11–17)^a^	13.14±1.56^b^	0.052^^^
**Menstrual cycles**			
**Regular**	95 (81.19%)	10 (71.42%)	0.106^#^
**Irregular**	13 (11.11%)	4 (28.57%)	
**NA**	9 (7.69%)	0 (0%)	
**Age at marriage (n = 112)**	20 (13–30)^a^	0	NA
**Duration of marriage (n = 112)**	28.23±12.09 (4–59)^b^	0	NA
**Total probable duration of illness (months, n = 108)**	111 (6–480)^a^	117.5±63.1^6^b	0.573^^^
**Total probable duration of illness (years, n = 108)**	9.25 (0.5–40)^a^	9.79±5.26^b^	0.573^^^
**Age of onset of disease (n = 108)**	37 (18–71)^a^	19.79±3.69^b^	<0.001^^^
**Comorbidity**	54 (46.15%)	2 (14.28%)	0.024^#^

* Chi-square test;

^ t-test;

# Fisher’s exact test,

$ Shapiro-Wilk test,

a Non-normal,

b Normal NA: not applicable; SSc: systemic sclerosis.

Among married women with SSc, the mean gravida was 2.26 ± 1.07 (range: 0–6; n = 264), indicating an average of just over two pregnancies per woman. The mean parity was 1.91 ± 0.97 (range: 0–5; n = 224). The mean number of abortions was 0.25 ± 0.54 (range: 0–2; n = 29), and MTPs were less frequent, with a mean of 0.09 ± 0.37 (range: 0–3; n = 11). The mean number of IUDs was very low (0.03 ± 0.18; range: 0–1; n = 4). The mean number of live births per woman was 1.89 ± 0.94 (range: 0–5; n = 221).

The pregnancy wastage ratio was 125 per 1000 pregnancies. The stillbirth rate was 18.09 per 1000 live births and 15.15 per 1000 pregnancies. The lifetime stillbirth rate was 34.18 per 1000 women, while the lifetime abortion rate was 247.86 per 1000 women, indicating a relatively higher occurrence of abortion compared to stillbirth in this cohort. The number of abortions per 1000 pregnancies was 109.84, showing that over 10% of pregnancies resulted in spontaneous or induced abortion. The CFR among married women was 1.88.

### Reproductive health outcomes before and after disease onset

Among the 117 married women with SSc, pregnancy outcomes were compared between those occurring before and after disease onset. A total of 251 pregnancies were reported prior to diagnosis (n = 110), whereas only 13 pregnancies occurred after disease onset (n = 10). The mean age at disease onset was significantly lower in women who became pregnant after diagnosis compared to those who conceived before disease onset (25.44 ± 5.83 years vs. 38.08 ± 10.42 years; P < 0.001). The mean age at marriage was comparable between the groups (21.10 ± 3.31 years vs. 20.37 ± 3.26 years; P = 0.502).

There were no significant differences in parity (P = 0.424), abortion rates (P = 0.641), or MTP rates (P = 0.432) between the two groups. Live birth rates were also comparable (P = 1.000). However, the mode of delivery differed significantly, with vaginal delivery being more frequent before disease onset (186 vs. 6; P = 0.023), while caesarean section (LSCS) was more common in post-disease pregnancies (4 out of 10 pregnancies) (**Table 2**).

**Table 2.** Comparison of fertility patterns and pregnancy outcomes before and after onset of SSc among married women.

**Parameters (n = 117)**	**Details of pregnancy prior to disease (n = 110)**	**Details of pregnancy after disease (n = 10)**	**P value**
**Gravida**	251	13	NA
**Age of onset of disease (gravida)**	38.08±10.42 (18 – 71)	25.44±5.83 (18 – 34)	<0.001^^^
**Age at marriage (n = 3616)**	20.37±3.26 (13 – 30)	21.1±3.31 (16 – 27)	0.502^^^
**Para**	214	10	0.424^#^
**Abortion**	27	2	0.641^#^
**MTP**	10	1	0.432^#^
**IUD**	4	0	NA
**Live birth**	210	11	1.000^#^
**Pregnancy wastage ratio**	123.5	153.84	NA
**Stillbirth per 1000 live births**	19.04	0	NA
**Number of stillbirths per 1000 pregnancies**	15.93	0	NA
**Lifetime abortion per 1000 females**	241.07	166.66	NA
**Number of abortions per 1000 pregnancy**	107.56	153.84	NA
**Cumulative fertility rate (CFR)**	1.87	0.91	NA
**Mode of delivery**			
**Vaginal**	186	6	0.023^#^
**LSCS**	23	4		
**Not specified**	5	0	

* Chi-square test;

^ t-test;

# Fisher’s exact test

NA: not applicable; SSc: systemic sclerosis; MTP: medical termination of pregnancy; IUD: intrauterine death; CFR: cumulative fertility rate; LSCS: lower segment caesarean section.

The most commonly used medications among the study participants were hydroxychloroquine, steroids, mycophenolate mofetil, and others, with significantly higher usage in the married group.

## DISCUSSION

The current study demonstrated that married women with SSc were significantly older at recruitment and had a higher mean age at disease onset compared to unmarried women. The earlier age at disease onset in unmarried women may indicate a more aggressive or early-onset phenotype in a subset of patients. Women with SSc experienced significant reproductive health challenges, including higher pregnancy wastage ratios, increased caesarean delivery rates, and poorer pregnancy outcomes compared to the general population. Pregnancy after disease onset was rare (13 pregnancies in 10 women), and these women had a significantly younger age at disease onset compared to those with pregnancies only before diagnosis. The mode of delivery differed markedly, with caesarean sections more frequent after disease onset, reflecting obstetric concerns about maternal or foetal complications in SSc.

The current study revealed a pregnancy wastage ratio of 125 per 1000 pregnancies, with higher abortion rates among pregnancies occurring after disease onset compared to those before onset (24.4% vs. 9.6%). The increased adverse pregnancy outcomes in SSc patients in the present study align with those of Kharbanda et al., who conducted a retrospective study of Indian women with SSc. They also found that pregnancies occurring after disease onset demonstrated a higher rate of maternal (OR 4.9) and foetal (OR 9.9) complications compared to those before onset. SSc patients had a higher abortion rate (OR 5.8), lower live birth rate, and required more frequent caesarean sections.^18^ A systematic review by Blagojevic et al. also reported increased frequencies of miscarriages, IUGR, preterm deliveries, and newborns with low birth weight.^19^ Similar trends were observed in the Italian multicentre IMPRESS study, which found significantly higher rates of preterm births (25% vs 12%), IUGR, and very-low birth weight infants among SSc patients compared to the general population.^8^ In contrast, Steen et al. reported that women with SSc can have acceptable pregnancy outcomes compared to controls, although they may still experience higher rates of premature births and small full-term infants.^20^

The current study reported a CFR of 1.88 among married women with SSc, which is lower than the national CFR of approximately 2.1 (National Family Health Survey-5, 2021) noted in the general population in India.^21^ This difference is likely attributable to self-selection, as women with SSc, particularly those diagnosed at a younger age, often avoided or delayed pregnancy to avoid potential maternal and foetal risks. In contrast, a retrospective Chinese study by Dai et al. reported that the infertility rate did not differ before and after disease onset.^22^ However, evidence from Chakravarty et al. supports the notion that women with SSc tend to avoid or postpone pregnancy post-diagnosis due to concerns over adverse maternal and foetal outcomes.^7^

SSc, being a disfiguring disease characterised by skin tightening of the face and hands, can affect physical appearance and hand function, thereby influencing social interactions and marriage prospects. In the present study, the mean age of onset among women who did not marry was significantly lower. Early disease onset appears to influence the overall fertility rate, but in this study, the primary attributable reason was the personal decision regarding childbearing. When the CFR was calculated only for married women who became pregnant after disease onset, it was significantly lower, possibly due to the direct biological effects of the disease. Other socio-economic factors may also contribute substantially to the decision not to conceive.

In the current study, the lifetime abortion rate among women with SSc was 247.9 per 1,000, over five times higher than the 47 per 1,000 reported nationally by Singh et al.^23^ Comparisons with regional data also reveal a marked difference, with rates of 14.7–16.9% reported in Varanasi (Agarwal et al.) and 8.6 per 1,000 pregnancies in Bihar (Kochar et al.)^24,25^ Similarly, the stillbirth rate in this cohort was 34.2 per 1,000, more than three times the 9.7 per 1,000 births documented in the National Family Health Survey.^26^ These disparities underscore the significantly higher burden of adverse obstetric outcomes in women with SSc compared to both national and regional population-based estimates.

Several other study findings have also corroborated that women with SSc have a higher risk of miscarriage (OR 1.6, 95% CI 1.22–2.22) and a significantly increased stillbirth rate compared with controls.^10,19,27^ In a study from Iran, successful pregnancy was reported in only 67% of post-disease-onset pregnancies, compared to 83.3% before onset (P = 0.01). Similarly, the frequency of preterm delivery increased from 4.3% to 16.3% after disease onset (P = 0.002).^28^ In a large nationwide analysis of over 11 million deliveries, Chakravarty et al. identified SSc in 504 cases and reported that, compared with the general obstetric population, affected women had a markedly increased risk of hypertensive disorders including preeclampsia (OR 3.71, 95% CI 2.25–6.15) and IUGR (OR 3.74, 95% CI 1.51–9.28), along with longer hospital stays.^7^ The study findings underscore the substantial adverse impact of SSc on reproductive outcomes and highlight the importance of preconception counselling and close perinatal monitoring in affected women.

Although there are studies, including those published in the *Mediterranean Journal of Rheumatology*, examining reproductive outcomes in autoimmune diseases, most have been conducted in Western or Middle Eastern cohorts.^29,30^ In contrast, data from low- and middle-income countries remain scarce. The present study is one of the first in the Indian context to compare reproductive outcomes before and after disease onset, as well as between married and unmarried women with SSc. It represents one of the earliest independent, prospective, multicentre observational efforts in India to systematically document reproductive health in this population using a standardised national registry (IRA database). The findings provide a critical evidence base for developing India-specific recommendations on reproductive counselling, fertility preservation, and antenatal monitoring in women with SSc. Given the heterogeneity in disease presentation and the strong influence of socio-cultural factors on reproductive decisions, such localised data are essential to support tailored patient counselling and optimise maternal-foetal outcomes.

Several previous studies have highlighted the need to address knowledge gaps among rheumatologists and to conduct regular fertility assessments in premenopausal women with SSc. A single-centre comparative study by Pecher et al. reported that anti-Muellerian hormone (AMH) concentrations, measured using electro-chemiluminescence immunoassay, were significantly lower in patients with SSc compared to controls (955 ng/L vs. 1,940 ng/L; p < 0.01). The authors emphasised the importance of routine AMH assessment in younger women with SSc to optimise pregnancy planning, fertility preservation, and treatment decisions.^31^ Similarly, a web-based survey among Indian rheumatologists, published in the *Mediterranean Journal of Rheumatology*, reported that many lacked confidence in discussing fertility preservation and assisted reproductive techniques. The study concluded that addressing these gaps, enhancing collaboration with obstetricians, and improving patient education could strengthen reproductive care in rheumatic diseases.^32^

This study has several strengths. It represents a multi-centre investigation involving 131 female patients with SSc recruited from seven rheumatology centres across India, enhancing the generalisability and robustness of the findings. The comprehensive assessment of reproductive parameters, including gravida, parity, CFR, and detailed pregnancy outcomes, adds significant depth to the analysis. Furthermore, subgroup comparisons between married and unmarried women, as well as pregnancies occurring before and after disease onset, provide valuable insights into the differential impact of SSc on reproductive trajectories. However, certain limitations should be acknowledged. The retrospective nature of data collection and partial reliance on patient recall may have introduced recall bias and limited the capture of information, particularly regarding drug use and comorbidities present before and after pregnancy. In addition, as the study population was recruited from specialised rheumatology centres, referral bias is possible.

These findings underscore the critical need for integrated, multidisciplinary care for women with SSc, encompassing timely preconception counselling, individualised risk stratification, and close maternal-foetal monitoring throughout pregnancy. As therapeutic advances continue to improve disease control and survival in SSc, reproductive health is emerging as an increasingly important component of comprehensive patient management.

## CONCLUSION

This multicentre analysis shows a substantial impact of the disease on reproductive health, with higher rates of pregnancy loss and altered delivery patterns. Married women have a later age at disease onset, a higher comorbidity burden, and greater lifetime exposure to pregnancies compared with unmarried women, while unmarried women report more menstrual irregularities. Pregnancies occurring after SSc onset are infrequent and tend to occur at a younger age, with a notable shift toward caesarean deliveries and reduced CFR. The high lifetime abortion rate and significant pregnancy wastage underscore the need for preconception counselling, individualised risk assessment, and close maternal-foetal surveillance. These findings provide important India-specific data to guide reproductive health management and counselling in women with SSc.

## Data Availability

Data will be provided on request.
