# Supplementary material for: Reproductive Outcomes and Fertility Patterns in Women with Systemic Sclerosis: A Multicentre Observational Study
Source: Mediterr J Rheumatol. 2025 Dec 31;36(4):566–72. doi: 10.31138/mjr.200825.ser (PMC12869440; doi:10.31138/mjr.200825.ser)
Supplement: Supplementary file 1 [file MJR-2025-0277_Supplementary-data.pdf]

**The following formulas were used to calculate various reproductive health metrics**

1. Pregnancy wastage ratio = (Total number of still births and abortions / Total number of pregnancies) * 1000

For both married and unmarried data, the calculation remains the same.

Pregnancy wastage ratio = (4+29)/264 *1000 = 125

The total number of stillbirths is 4, and the total number of abortions is 29. The total number of pregnancies is 264, which is considered for calculation.

2. Still births per 1000 live births = (Total number of stillbirths/Total number of live births) *1000

For both married and unmarried data, the calculation remains the same.

Still births per 1000 live births = (4/221) *1000 = 18.09

The total number of stillbirths is 4, and the total number of live births is 221, considered for calculation.

3. Number of Stillbirths per 1000 pregnancy = (Total number of stillbirths / Total number of pregnancies) * 1000.

For both married and unmarried data, the calculation remains the same.

Number of Stillbirths per pregnancy = (4 / 264) * 1000 = 15.15

The total number of stillbirths is 4, and the total number of pregnancies is 264, considered for calculation.

4. Lifetime Stillbirth rate per 1000 women = (Total number of Stillbirths / Total number of women) * 1000.

For married data

Lifetime Stillbirth rate per woman = (4 / 117) *1000 = 34.18

In this case, 117 married women were considered in the data set. Among these women, 4 stillbirths are used to calculate the Lifetime Stillbirth rate per woman.

For both married and unmarried data.

Lifetime Stillbirth rate per woman = (4 / 131) *1000 = 30.53

In this case, 117 married and 14 unmarried women were considered, resulting in a total of 131 women in the data set. Among these, there are 4 instances of stillbirths, which are used to calculate the Lifetime Stillbirth rate per woman.

5. Number of abortions per 1000 pregnancy = (Number of abortions / Number of pregnancy) *1000

For both married and unmarried data, the calculation remains the same.

Number of abortion per pregnancy = (29 / 264) = 109.84

Number of abortions = 29

6. Lifetime abortions per 1000 females = (Number of abortions / Total number of women) *1000

For married data

Abortion per female = (29 / 117) *1000 = 247.86

Number of abortions = 29

The total number of women, 117, in the married data is used to calculate the lifetime abortion rate per 1,000 females.

7. Cumulative Fertility Rate (CFR) = Number of live births / Total number of women

For married data

Fertility index (TFR) = 221 / 117 = 1.88

In this case, 117 married women were considered in the data set. Among these women, 221 live births are used to calculate the Cumulative Fertility Rate (CFR).

8. Lifetime abortions per 1000 females = (Number of abortions / Total number of women) *1000

For both married and unmarried data.

Number of abortions per female = (29 / 131) *1000 = 221.37

In this case, 117 married and 14 unmarried women were considered, resulting in a total of 131 women in the data set. Among these, there are 29 instances of abortion, which are used to calculate the number of lifetime abortions per 1000 females.

9.Cumulative Fertility Rate (CFR) = Number of live births / Total number of women

For both married and unmarried data.

Cumulative Fertility Rate (CFR) = 221 / 131 = 1.68

In this case, 117 married and 14 unmarried women were considered, resulting in a total of 131 women in the data set. Among these women, 221 live births are used to calculate the Cumulative Fertility Rate (CFR).

10. Pregnancy wastage ratio = (Total number of still births and abortions / Total number of pregnancies) * 1000

Prior to disease

Pregnancy wastage ratio = (4+27)/251 *1000 = 123.50

The total number of stillbirths is 4, and the total number of abortions is 27. The total number of pregnancies is 251, considered for calculation.

After the disease

Pregnancy wastage ratio = (0+2)/13 *1000 =153.84

The total number of stillbirths is 0, and the total number of abortions is 2. The total number of pregnancies is 13, considered for calculation.

11. Stillbirth per 1000 live births = (Total number of still births / Total number of live births) *1000

Prior to disease

Still births per 1000 live births = (4/210)*1000 = 19.04

The total number of stillbirths is 4, and the total number of live births is 210, considered for calculation.

After the disease

Still births per 1000 live births = (0/11) *1000 = 0

The total number of stillbirths is 0, and the total number of live births is 11, considered for calculation.

12. Number of Stillbirths per 1000 pregnancy = (Total number of stillbirths / Total number of pregnancies) *1000.

Prior to disease

Number of Stillbirths per pregnancy = (4 / 251) *1000 = 15.93

Total number of stillbirths prior to disease is 4, and the total number of pregnancies prior to disease is 251, considered for calculation.

After the disease

Number of Stillbirths per pregnancy = (0 / 13) *1000 = 0

Total number of stillbirths after disease is 0, and the total number of pregnancies after disease is 13, considered for calculation.

13. Lifetime abortion per 1000 females = (Number of abortions / Total number of women) *1000

Prior to disease

Number of abortions per female = (27 / 112) *1000 = 241.07

Out of 112 patients, 105 had pregnancies prior to the disease, 5 had pregnancies before and after the disease, while 2 did not have any pregnancies before or after the disease. Among these patients, there were 27 abortions considered for calculation.

After the disease

Number of abortions per female = (2 / 12) *1000 = 166.66

Out of 12 patients, 5 had pregnancies after the disease, 5 had pregnancies before and after the disease, while 2 did not have any pregnancies before or after the disease. Among these patients, there were 2 abortions considered for calculation.

14. Number of abortions per 1000 pregnancy = (Number of abortions / Number of pregnancy) *1000

Prior to disease

Number of abortion per pregnancy = (27 / 251) *1000 = 107.56

The number of abortions prior to the disease is 27. A total of 251 pregnancies are considered to calculate the number of abortions per pregnancy.

After the disease

Number of abortion per pregnancy = (2 / 13)*1000 = 153.84

The number of abortions after the disease is 2. A total of 13 pregnancies are considered to calculate the number of abortions per pregnancy.

15. Cumulative Fertility Rate (CFR) = Number of live births / Total number of women

Prior to disease

Cumulative fertility rate (CFR) = 210 / 112 = 1.875

Out of 112 patients, 105 had pregnancies prior to the disease, 5 had pregnancies before and after the disease, while 2 did not have any pregnancies before or after the disease. Among these patients, 210 live births were considered for calculation.

After the disease

Completed Fertility Rate (CFR) = 11 / 12 = 0.91

Out of 12 patients, 5 had pregnancies after the disease, 5 had pregnancies before and after the disease, while 2 did not have any pregnancies before or after the disease. Among these patients, 11 live births were considered for calculation.

## References

### Cumulative fertility rate


1. Manual X - Indirect Techniques for Demographic Estimation | Population Division [Internet]. [cited 2025 Aug 14]. Available from: https://www.un.org/development/desa/pd/content/manual-x-indirect-techniques-demographic-estimation


### Number of Stillbirths per 1000 pregnancies


- **World Health Organization (WHO):** Provides global health statistics and definitions related to stillbirths and maternal health. Their reports and guidelines often include methods for calculating and interpreting stillbirth rates.

Maternal, Newborn, Child and Adolescent Health and Ageing [Internet]. [cited 2025 Aug 14]. Available from: https://www.who.int/teams/maternal-newborn-child-adolescent-health-and-ageing

- **Research Articles:** Peer-reviewed articles often provide case studies and methodologies for calculating stillbirth rates.
- You can search databases like PubMed, Google Scholar, or specific journals like "The Lancet" or "BJOG: An International Journal of Obstetrics & Gynaecology."

- **Centers for disease control and prevention (CDC):** Offers resources and statistical data on stillbirths and perinatal health.
  CDC. Stillbirth. 2025 [cited 2025 Aug 14]. About Stillbirth. Available from: https://www.cdc.gov/stillbirth/about/index.html
- **Books and Academic Journals:**
  "Textbook of Perinatal Medicine" by R. R. de Vries and G. S. Weiner

- "**Obstetrics and Gynecology"** by David M. L. and E. G. K. Edsall, which provides detailed chapters on pregnancy outcomes, including stillbirths.
- **Lifetime Stillbirth rate per woman**
- **World Health Organization (WHO)**
- **Provides global and regional statistics on stillbirths, including methods for calculating lifetime rates.**
  Maternal, Newborn, Child and Adolescent Health and Ageing [Internet]. [cited 2025 Aug 14]. Available from: https://www.who.int/teams/maternal-newborn-child-adolescent-health-and-ageing

- **Centers for Disease Control and Prevention (CDC)**
- **Offers information on stillbirth statistics and health guidelines.**
  CDC. Stillbirth. 2025 [cited 2025 Aug 14]. About Stillbirth. Available from: https://www.cdc.gov/stillbirth/about/index.html

- **Number of abortions per pregnancy**
- **World Health Organization (WHO)**
- **The WHO provides global and regional statistics on abortion and related reproductive health metrics.**

Abortion care guideline [Internet]. [cited 2025 Aug 14]. Available from: https://www.who.int/publications/i/item/9789240039483

- Centers for Disease Control and Prevention (CDC)
- Offers information on abortion statistics and related health data.

CDC. Reproductive Health. 2024 [cited 2025 Aug 14]. Abortion Surveillance Findings and Reports. Available from: https://www.cdc.gov/reproductive-health/data-statistics/abortion-surveillance-findings-reports.html

- **Abortions per female**
- **World Health Organization (WHO)**
- **Offers comprehensive data and guidelines on reproductive health, including abortion rates and statistics.**

- Abortion care guideline [Internet]. [cited 2025 Aug 14]. Available from: https://www.who.int/publications/i/item/9789240039483

- **Centers for Disease Control and Prevention (CDC)**
- **Provides information and statistical data on abortions and reproductive health.**
- CDC. Reproductive Health. 2024 [cited 2025 Aug 14]. Abortion Surveillance Findings and Reports. Available from: https://www.cdc.gov/reproductive-health/data-statistics/abortion-surveillance-findings-reports.html
